# Interaction of NMDA Receptor and Pacemaking Mechanisms in the Midbrain Dopaminergic Neuron

**DOI:** 10.1371/journal.pone.0069984

**Published:** 2013-07-19

**Authors:** Joon Ha, Alexey Kuznetsov

**Affiliations:** 1 Laboratory of Biological Modeling, The National Institute of Diabetes and Digestive and Kidney Diseases (NIDDK), National Institute of Health, Bethesda, Maryland, United States of America; 2 Department of Mathematical Sciences and Center for Mathematical Biosciences, Indiana University, Purdue University Indianapolis, Indianapolis, Indiana, United States of America; Neuroscience Campus Amsterdam, VU University, The Netherlands

## Abstract

Dopamine neurotransmission has been found to play a role in addictive behavior and is altered in psychiatric disorders. Dopaminergic (DA) neurons display two functionally distinct modes of electrophysiological activity: low- and high-frequency firing. A puzzling feature of the DA neuron is the following combination of its responses: N-methyl-D-aspartate receptor (NMDAR) activation evokes high-frequency firing, whereas other tonic excitatory stimuli (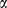-amino-3-hydroxyl-5-methyl-4-isoxazolepropionate receptor (AMPAR) activation or applied depolarization) block firing instead. We suggest a new computational model that reproduces this combination of responses and explains recent experimental data. Namely, somatic NMDAR stimulation evokes high-frequency firing and is more effective than distal dendritic stimulation. We further reduce the model to a single compartment and analyze the mechanism of the distinct high-frequency response to NMDAR activation vs. other stimuli. Standard nullcline analysis shows that the mechanism is based on a decrease in the amplitude of calcium oscillations. The analysis confirms that the nonlinear voltage dependence provided by the magnesium block of the NMDAR determine its capacity to elevate the firing frequency. We further predict that the moderate slope of the voltage dependence plays the central role in the frequency elevation. Additionally, we suggest a repolarizing current that sustains calcium-independent firing or firing in the absence of calcium-dependent repolarizing currents. We predict that the ether–a-go-go current (ERG), which has been observed in the DA neuron, is the best fit for this critical role. We show that a calcium-dependent and a calcium-independent oscillatory mechanisms form a structure of interlocked negative feedback loops in the DA neuron. The structure connects research of DA neuron firing with circadian biology and determines common minimal models for investigation of robustness of oscillations, which is critical for normal function of both systems.

## Introduction

The central dopamine system is involved with many behavioral and cognitive tasks [1]. Dopamine neurotransmission is found to play a role in addictive behavior [2] and is altered in psychiatric disorders [3]. The DA neuron fires a burst of activity whenever an animal receives an unpredicted reward or a reward-predicting stimulus [4]. The mechanisms underlying the excitability of the DA neuron have attracted the interest of electrophysiologists for many years. One of the major questions is what synaptic input triggers the excitation, or is a combination of inputs required? The stereotypic response of the DA neuron to the unpredicted reward is a burst of activity with firing frequencies above 20 Hz. In vivo, the bursts are singular events superimposed on a low-frequency single-spiking background firing (1–5 Hz). A burst is shown to be triggered by the stimulation of glutamatergic inputs, of which NMDA, but not (AMPA) receptor activation was found to be critical [5]–[7]. *In vitro,* these distinct responses to the activation of the two types of glutametergic synapses are confirmed: bursts evoked by stimulations in slices are dependent on the activation of NMDA receptors, whereas tonic activation of AMPA receptors alone does not elicit bursting [8], [9]. This is a puzzling feature of the DA cells that distinguishes it from many other types of neurons: How does DA neurons differentiate the NMDAR and AMPAR stimulation?

Similar to the AMPAR stimulation, tonic somatic current injections cannot elevate the firing frequency significantly: Applied depolarization blocks firing in the DA neuron as the frequency increases above 10 Hz [10], [11]. Interestingly, somewhat higher frequencies are possible during a transient after the onset of the current injection [10], [11], but not after the onset of a tonic AMPAR stimulation [9], which evokes only a single spike. These low frequencies and the susceptibility to the blockade of firing are features that distinguish the DA neuron.

Our previous model [12] has suggested how the neuron can respond differentially to the applied current and AMPA vs. NMDA receptor stimulation. Several laboratories tested our major predictions, and found one of them inconsistent with the new experimental data [9], [11], [13]. Namely, the false prediction was the dendritic drive for the high-frequency firing: Because of the difference in morphology, the isolated dendrites must have a much higher natural frequency. We had found how parts of the neuron can dominate its dynamics and, consequently, determine the frequency. To change its frequency, the neuron needs only a stimulus that switches the dominance from one part of the neuron to another. We had shown that NMDAR switches the dominance from the low-frequency soma to high-frequency dendrites in the model. However, the switch in dominance required distribution of the NMDA stimulation onto fine dendrites, whereas somatic NMDA stimulation was unable to elevate the frequency. The new experiments have demonstrated the opposite - somatic NMDAR stimulation or a virtual current elicits high-frequency firing [9], [13]. This disproves our mechanism.

In this paper, we design a new mechanism for the differential responses of the DA neuron to applied depolarization and AMPA vs. NMDA receptor activation. The major distinction with the old mechanism is that the gradient of the natural frequencies along the dendrites is not used. First, this allowed us to achieve the high frequencies either for somatic or dendritic, as well as whole neuron NMDAR stimulation. Consistent with experiments [11], a proximal or somatic stimulation is more efficient in the model. Second, it allowed for the major reduction of the model to only one compartment. Based on this reduction, we give a very simple explanation of the frequency elevation. Remarkably, the biophysical properties described by the model are exactly the same as in the previous one [12]. However, the calibration of the model is different, and the most important is an altered voltage dependence of the NMDAR current. Consistent with experiments [9], [13], our study highlights that the voltage dependence provided by the magnesium block of the NMDAR current is critical for the elevation of the frequency. We further predict that, of the parameters of the NMDAR current, the moderate slope of its voltage dependence is central in the new mechanism. The results refocus the attention of the experimentalists from the search of new determinants of the burst firing to the well-known oscillatory mechanism in the light if its new interaction with the NMDAR current.

Another surprising feature of the DA neuron accentuated recently is its calcium-independent firing: the neurons continue robust firing at a low pacemaking rate after pharmacological blockade of Ca^2+^ channels or Ca^2+^ chelation, or in Ca^2+^-free solution [11], [14]–[18]. How can a neuron display calcium-independent firing if its oscillatory mechanism is consistently shown to rely on the calcium and calcium-dependent potassium currents (see e.g. [19]–[21])? This evoked a vivid discussion about the contribution of calcium vs. sodium channels to the depolarization phase of the voltage oscillations and resulted in the conclusion that both types of channels contribute to the same oscillatory mechanism [22] and that calcium influx is not essential for oscillations [13]. What receives no attention in these papers is that something must repolarize the membrane when the calcium-dependent potassium current does not activate. This cannot be a strong delayed rectifier potassium current because this current would support a high frequency indifferently to the type of stimulation: applied current, AMPA or NMDA receptor stimulation.

We suggest a voltage-dependent potassium channel that supports oscillations when the calcium-dependent repolarization mechanisms do not activate. No current had been suggested for this role, and we start with the assumption-free basic potassium current and calibrated it according to its function of supporting oscillations with particular characteristics. Then, we identify the current as the ether-a-go-go (ERG) current. ERG1 proteins are expressed in the substantia nigra pars compacta neurons [23]. Experiments show that the ERG current is functional in the DA neuron [24], [25]. The new current allows us to reproduce another recent result - the high-frequency firing persists under an SK current blocker apamin [9]. We study the interaction between the repolarizing currents and suggest how calcium-dependent and calcium-independent oscillatory mechanisms are combined in the DA neuron. The currents form a structure in which two feedback loops share components, and we adopt the term of interlocked feedback loops from another discipline, circadian biology for our new representation of the DA neuron. This establishes a common framework for understanding robustness and regularity of oscillations critical in both disciplines.

## Materials and Methods

We build a reduced model of the DA neuron to understand its properties rather than a detailed biophysical model. Thus, only currents that are shown to be critical for oscillations are included. The structure of the model is similar to that in our previous publication [12]. The major changes are altered NMDA voltage dependence and a newly introduced ERG-type voltage-dependent potassium current. The model is presented in two morphologies: (1) a reconstruction of a DA neuron and (2) a single compartment that ignores the spatial structure of the neuron. We show that these two morphologies display very similar patterns. The biophysical mechanisms included in both morphologies are the same. They are described below.

### Biophysical Properties

Both one-compartment and reconstructed morphology models studied below implement the following membrane mechanisms. The central currents in the model are an L-type non-inactivating voltage-dependent calcium current, an SK-type Ca^2+^-dependent potassium current, and a voltage-dependent potassium current, calibration of which suggests that it’s an ERG-type current. Gating of the calcium current is fast, and it is treated as instantaneous for simplicity. Gating of the Ca^2+^-dependent potassium current is also much faster than the timescale of its gating variable, Ca^2+^ concentration. Thus, we omit an extra gating variable for this current, treating it as instantaneous as well. By contrast, gating of the ERG current requires an explicit gating variable for generating oscillations under blockade of the Ca^2+^-dependent current. We calibrate it to play this role and discuss its parameters as a model prediction.

The four resulting equations are:
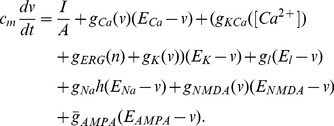
(1)

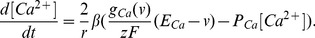
(2)

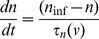
(3)

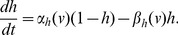
(4)


Here, *v* is voltage, 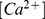 is calcium concentration, *n* is the gating (activation) variable of the ERG current, and *h* is the inactivation gating variable for the fast sodium current. In the voltage equation (1), 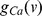 is a voltage-dependent Ca^2+^ conductance; 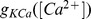 is a Ca^2+^-dependent K^+^ conductance; 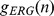 is the ERG conductance. A small leak conductance 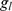 and a voltage-gated instantaneous potassium conductance 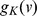 are included to limit the input resistance and to confine the voltage in the subthreshold range, respectively. We include a spike-producing fast sodium current with maximal conductance 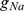. The current was found not to be essential for generating either high- or low-frequency activity [9], [20]. Thus, we calibrated the model to display firing patterns similar to the subthreshold oscillations when the fast sodium current is blocked.

Gating for the fast sodium current is in the standard Hodgkin-Huxley form. The activation of the current is assumed instantaneous and described by the function.
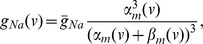
where



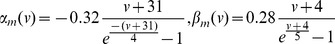



The inactivation kinetics is described by Eq. (4), where.
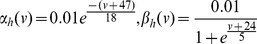



The current is calibrated to produce a spike per each maximum of the subthreshold oscillations without qualitatively changing the oscillatory pattern.

**Figure 1:**
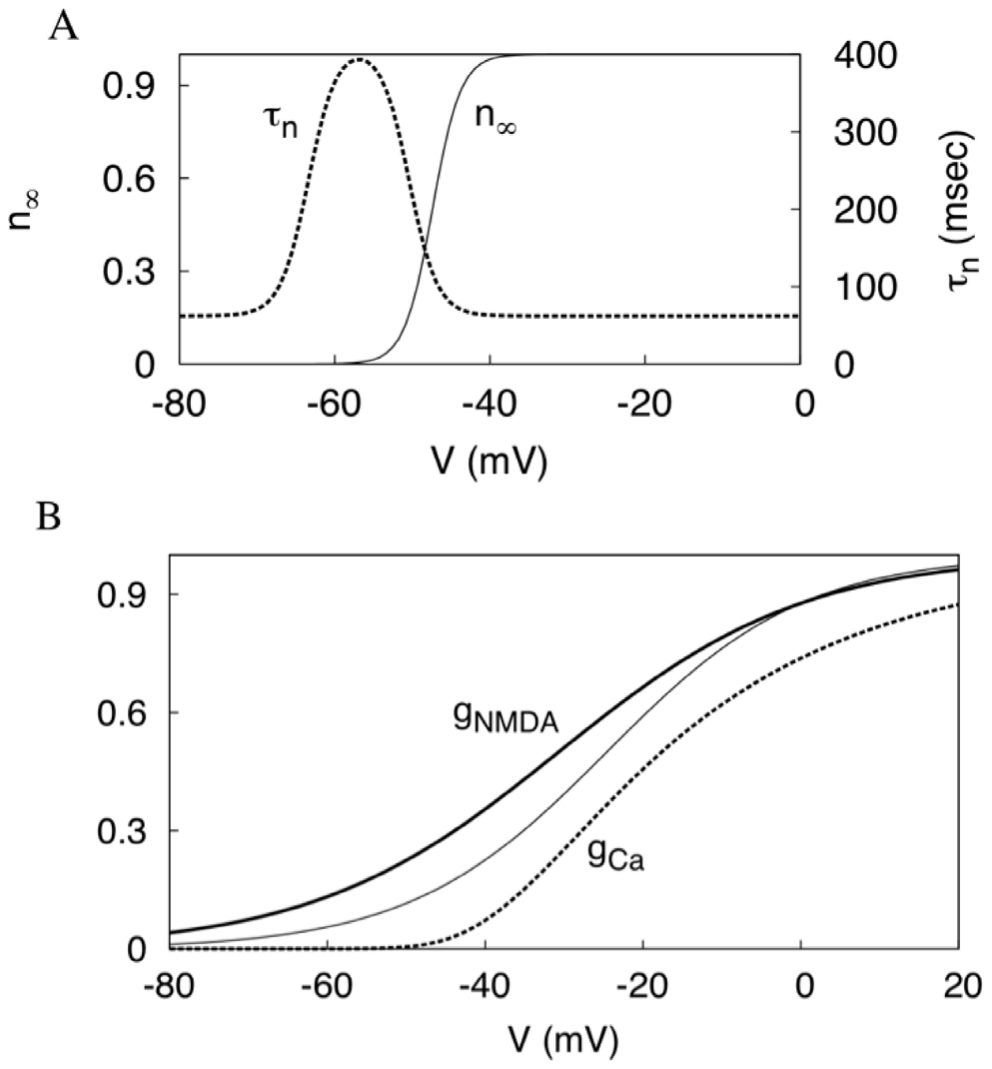
Model calibration. (A) The activation curve and the time constant of the ERG current. (B) The comparison of the activation curves for the NMDAR conductance in the present (solid bold) and the previous (solid thin) models, and the calcium current (dashed). All conductances are normalized to the maximum value of 1.

The equation for the gating variable of the ERG current (3) is in the standard Hodgkin-Huxley form with the following activation function and time constant:
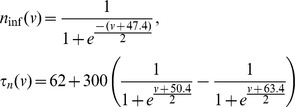



The functions are shown in Fig. 1A, and their parameters are a product of calibration of this current to sustain pacemaking in the absence of the Ca^2+^-dependent potassium current. The properties of this pacemaking (see below) allow us to specifically determine the half-activation of this current, while the time constant is determined qualitatively rather than quantitatively. The parameter search was performed manually because the criteria are hard to automate.

Conductances of the currents in Eq. (1) are given by the following functions:
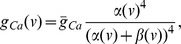
where,



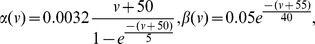


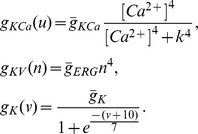



Here, all maximal conductances are given per unit of surface area in 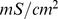 (see Table S1 for the values of the parameters). The voltage dependence of the Ca^2+^ current replicates the characteristic low-threshold L-type current found in DA neurons [26], [27]. The calcium current is shown to be non-inactivating [20]. The fourth power dependence on Ca^2+^ concentration above is typically used to best represent the characteristics of the SK type Ca^2+^-dependent potassium current [28]. We choose its parameters in the range estimated for the DA neuron [20].

The additive parameter *I* in Eq. (1) represents an applied current (in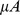). It is normalized by the surface area of the neuron *A* (in 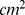). The conductance 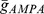 is a constant conductance density of a linear AMPAR synaptic current. The nonlinear function of the voltage with 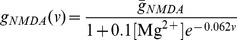 reflects the activation of the NMDAR synaptic current (see Fig. 1B). Here, 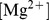 is magnesium concentration, and the magnesium block is treated as instantaneous. The parameters of this voltage dependence are very close to the values previously used in the literature [12], [29]. An important modification is a decrease in the slope of the NMDA conductance activation 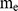 from 0.08 to 0.062. The parameter stays in the range measured experimentally [30], [31]. Interestingly, the more gradual dependence is also used in dynamic clamp experiments [9], [13]. As shown below, the modification is very important because it is responsible for the high frequency firing achieved during somatic NMDA receptor activation.

The calcium equation (2) represents balance between Ca^2+^ entry via the L current and Ca^2+^ removal via a pump. After entering the cell, Ca^2+^ binds to a buffering protein. Buffering is assumed instantaneous and is taken into account by multiplying by the buffering coefficient 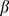. This coefficient is the ratio of free to total intracellular Ca^2+^. Therefore, [Ca^2+^] represents *free* intracellular Ca^2+^ concentration, and the parameters were estimated before in experiments [20].

### Morphologies

The biophysical mechanisms (currents and pumps) described above were inserted into two different morphologies: a single compartment, and a reconstruction of a DA neuron [20]. The equations introduced above fully describe the one-compartmental model. Computer simulations of this model were done using XPPAUT [32].

Simulations in the reconstructed morphology [20] were done using NEURON (http://www.neuron.yale.edu). All biophysical mechanisms were distributed homogeneously along the dendrites and in the soma. The dendrites and soma were divided into 50 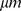-long equipotential segments. The value of 60 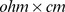 was used for the cytoplasmic resistivity, which is in the range of previously used (see e.g. [33]).

The models are published online in Model-DB.

## Results

### Reconstructed Morphology Model

#### Somatic NMDAR activation evokes high-frequency firing and subthreshold oscillations

**Figure 2:**
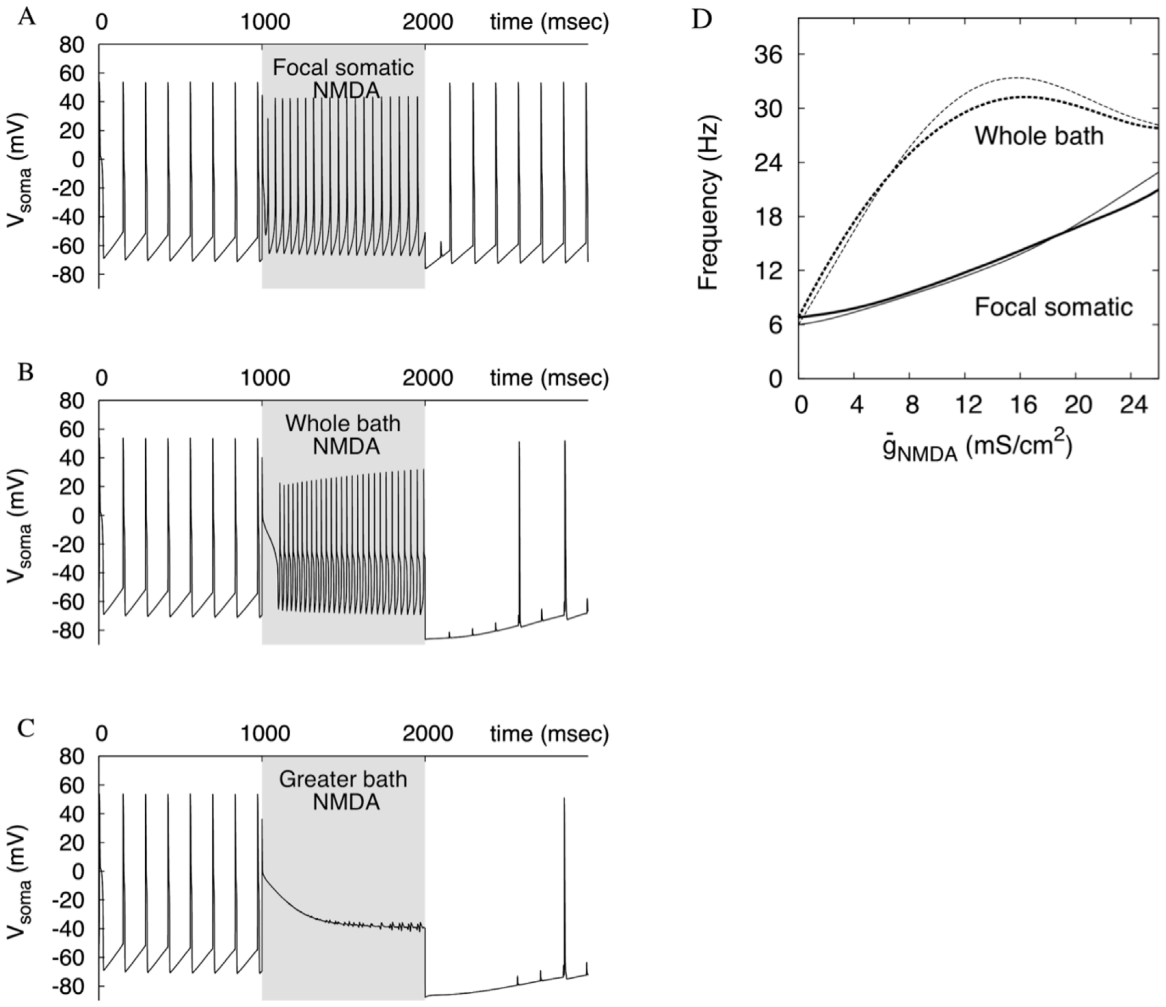
Somatic NMDA receptor activation effectively elevates the frequency. (A) NMDA is activated only in the soma for 1 sec (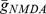 = 26 mS/cm^2^). The frequency rises to 25 Hz in response. (B) Simulated whole bath application of NMDA agonist evokes yet higher frequency than the focal somatic (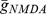 = 26 mS/cm^2^). (C) Excessive NMDA activation blocks the oscillations (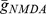 = 39 mS/cm^2^). (D) The dependence of the frequency on the maximal conductance of the NMDA current. The thin curves are for the model without the fast sodium current. A minimum amplitude of 5 mV is set for all calculations of the frequency in order to exclude the small amplitude oscillations.

Experiments [9], [13] show that NMDA receptor activation restricted to the soma effectively evokes high-frequency oscillations. However, modeling reproduced frequency elevation under dendritic, but not somatic, NMDA synaptic stimulation [12]. We correct this discrepancy: Fig. 2 A shows the effect of simulated somatic NMDA receptor activation implemented in a reconstructed morphology of the DA neuron. The NMDAR current is injected into the soma at 1000 ms for the duration of 1000 ms. The activation and removal of the current are modeled as instantaneous step functions and cause rapid transition between high- and low-frequency oscillations. The frequency reaches 25 Hz. A similar frequency increase occurs if a dendritic, but not somatic, or both the dendritic and somatic NMDA receptors are activated (Fig. 2B, D). An excessive NMDAR conductance results in depolarization block (Fig. 2 C). For the whole-neuron NMDAR activation (bath NMDA), the frequency above 20 Hz (typical for *in vivo* bursts) is achieved at much lower NMDAR conductance levels (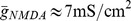). These levels are well within the physiological range. Several times higher NMDAR conductance is necessary to achieve that frequency for the soma-only excitation (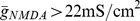). This is consistent with experiments [13].

**Figure 3:**
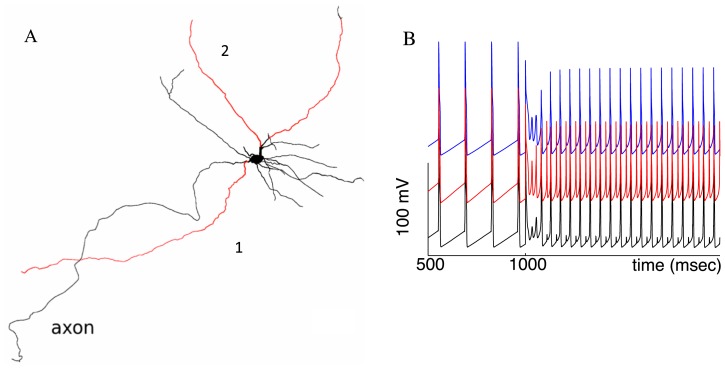
Achieving high frequency requires simultaneous NMDA receptor stimulation of distal dendrites. (A) The dendrites receiving NMDA stimulation are marked in read (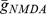 = 14 mS/cm^2^). (B) Firing a somatic spike (black) requires simultaneous firing of the three dendrites. The membrane potential for the dendrites 1 and 2 are shown in red and blue respectively. A spike in dendrite 1 alone evokes only a small spikelet in the soma.

The frequency rise depends on the spread of NMDA receptor activation as well as the focus of its application. We simulated iontophoresis of an NMDAR agonist to the distal dendrites of the neuron. No high frequency was achieved when any single branch of the dendritic tree was stimulated. The oscillations took a complex form of intermixed singular high- and multiple low-amplitude oscillations (mixed mode; data not shown). The total surface area of the stimulated dendrite was similar to, or even greater than the somatic one. Only when three or more distal dendrites were stimulated together (Fig. 3A, red), did the frequency rise significantly. Compared to the soma, approximately three times greater area of distal dendrites had to be affected by NMDA to evoke the high frequency. This is because distal dendrites need to synchronize across the dendritic tree for their concerted influence to dominate the soma and proximal dendrites (Fig. 3B). When there are stimuli that partition the dendritic tree, it is no longer synchronized. In Fig. 3B, even the dendrites that receive the NMDAR stimulation are not synchronized one-to-one: the dendrite 1 files an extra spike per the period (red trace). This spike is not enough to evoke a spike in the soma, and only when all three dendrites fire, the somatic spike follows. No further increase in the NMDAR conductance synchronizes the dendrites one-to-one. As a result, distal dendritic NMDAR stimulation is less effective than focused proximal or somatic.

**Figure 4:**
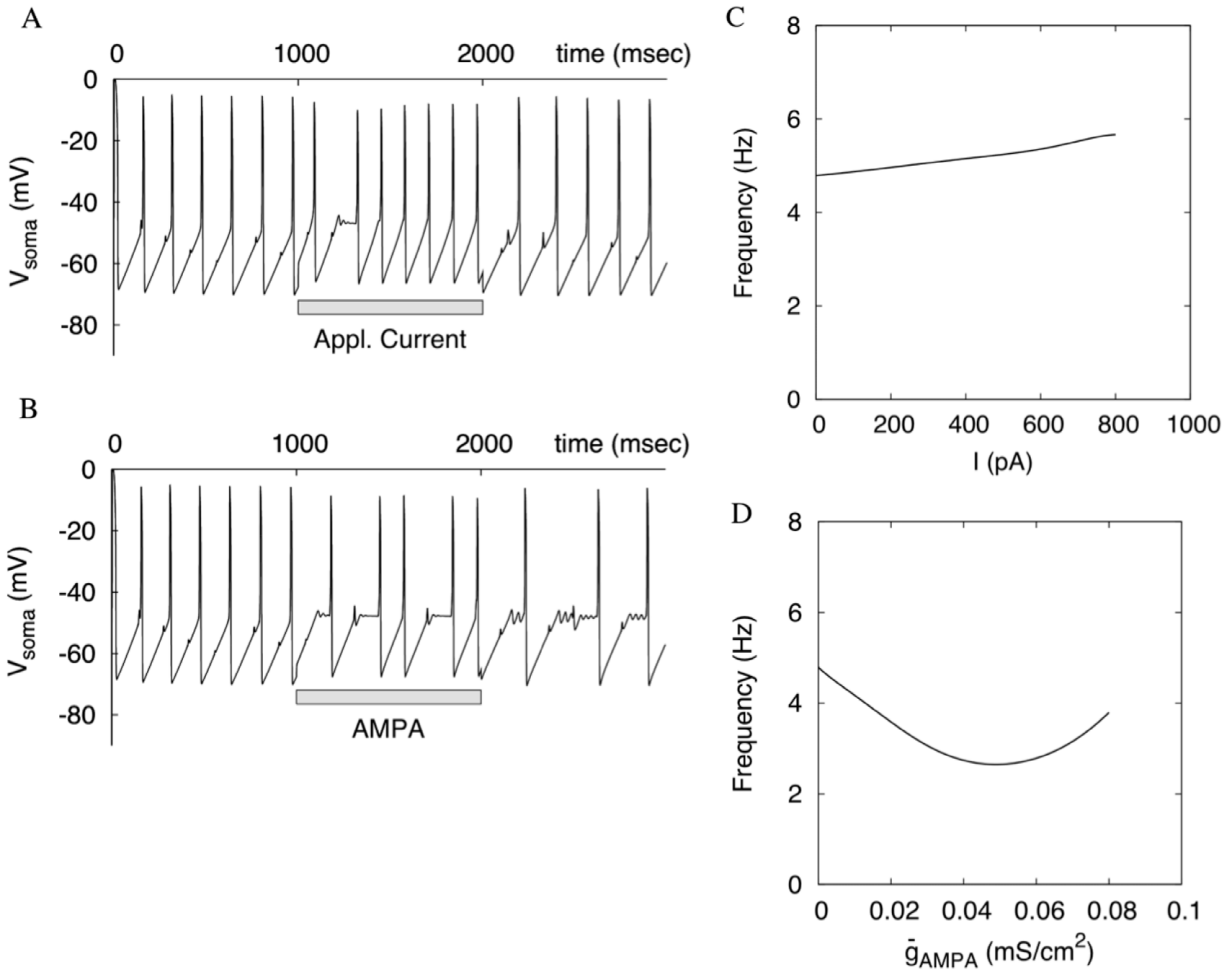
Tonic applied depolarization and AMPA receptor activation are unable to significantly elevate the frequency. (A) An applied current is injected into the soma for 1000 ms at an intensity slightly lower than that causing blockade of oscillations (700 pA). (B) Tonic AMPA receptor stimulation causes complex oscillations and, consequently, only reduces the frequency. (C) and (D) The dependence of the frequency on the applied current and AMPA maximal conductance, respectively. The oscillations are blocked at the end of each curve.

**Figure 5:**
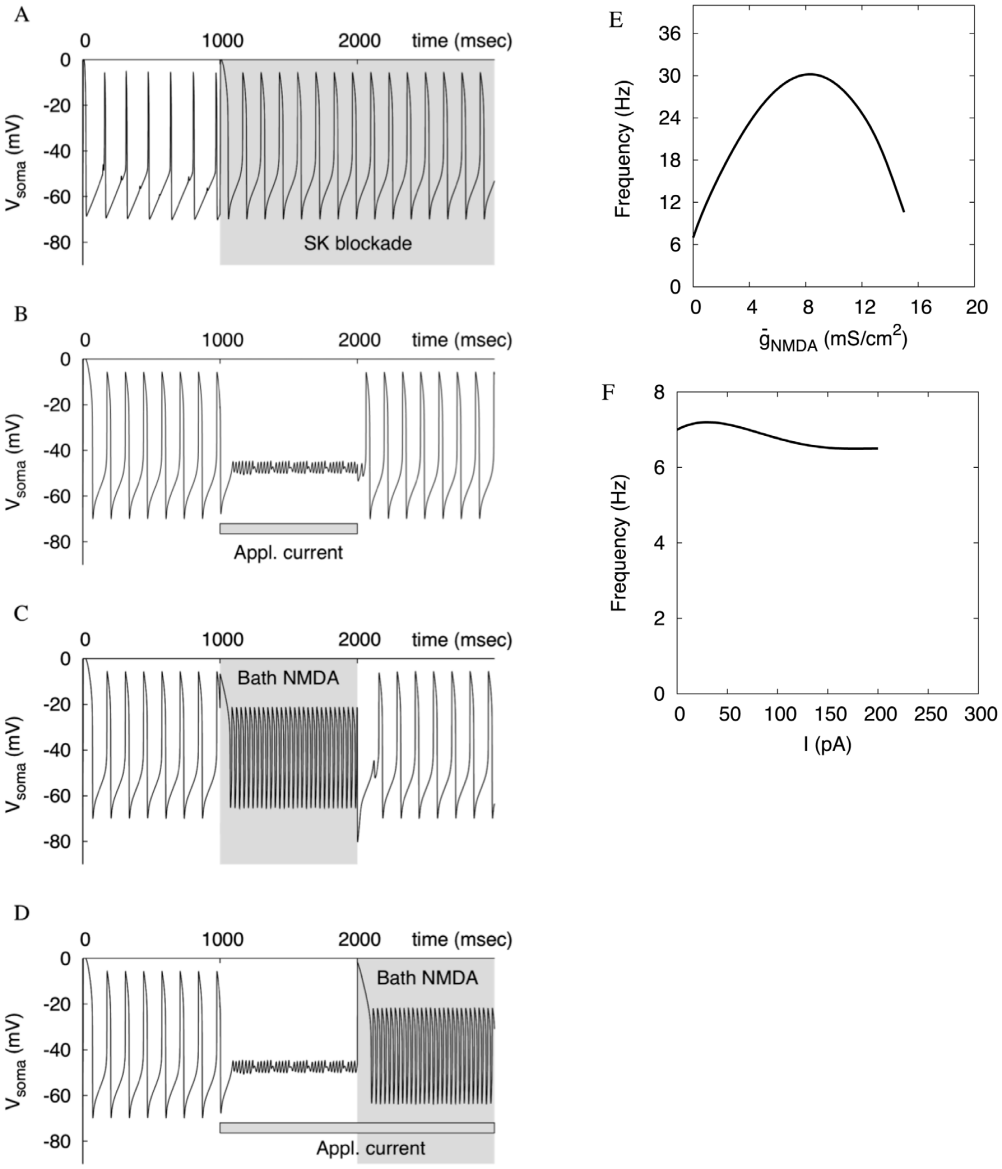
Oscillations persist under SK current blockade. (A) The blockade moderately increases the frequency of oscillations. (B) A very weak applied depolarization (320 pA) is enough to block the oscillations. (C) NMDA receptor activation elevates the frequency as effectively as in the presence of the SK current. (D) A moderate NMDAR activation rescues the neuron from the blockade of oscillations caused by applied depolarization. (E,F) The dependence of the frequency on the NMDAR conductance and the applied current. The plots are similar to Fig. 2D and Fig. 4C respectively, but both horizontal scales are expanded and depolarization block occurs at lower values than with the SK current.

**Figure 6:**
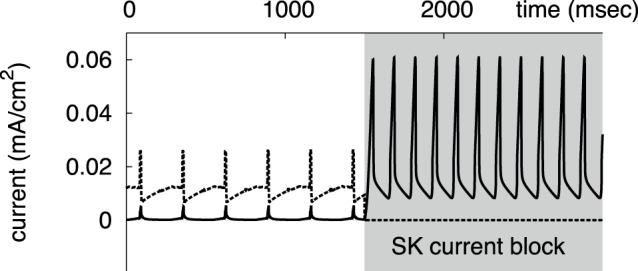
The ERG (solid line) and SK (dashed line) currents at the onset of the SK current blockade. The activation of the ERG current is much stronger when the SK current is blocked.

**Figure 7:**
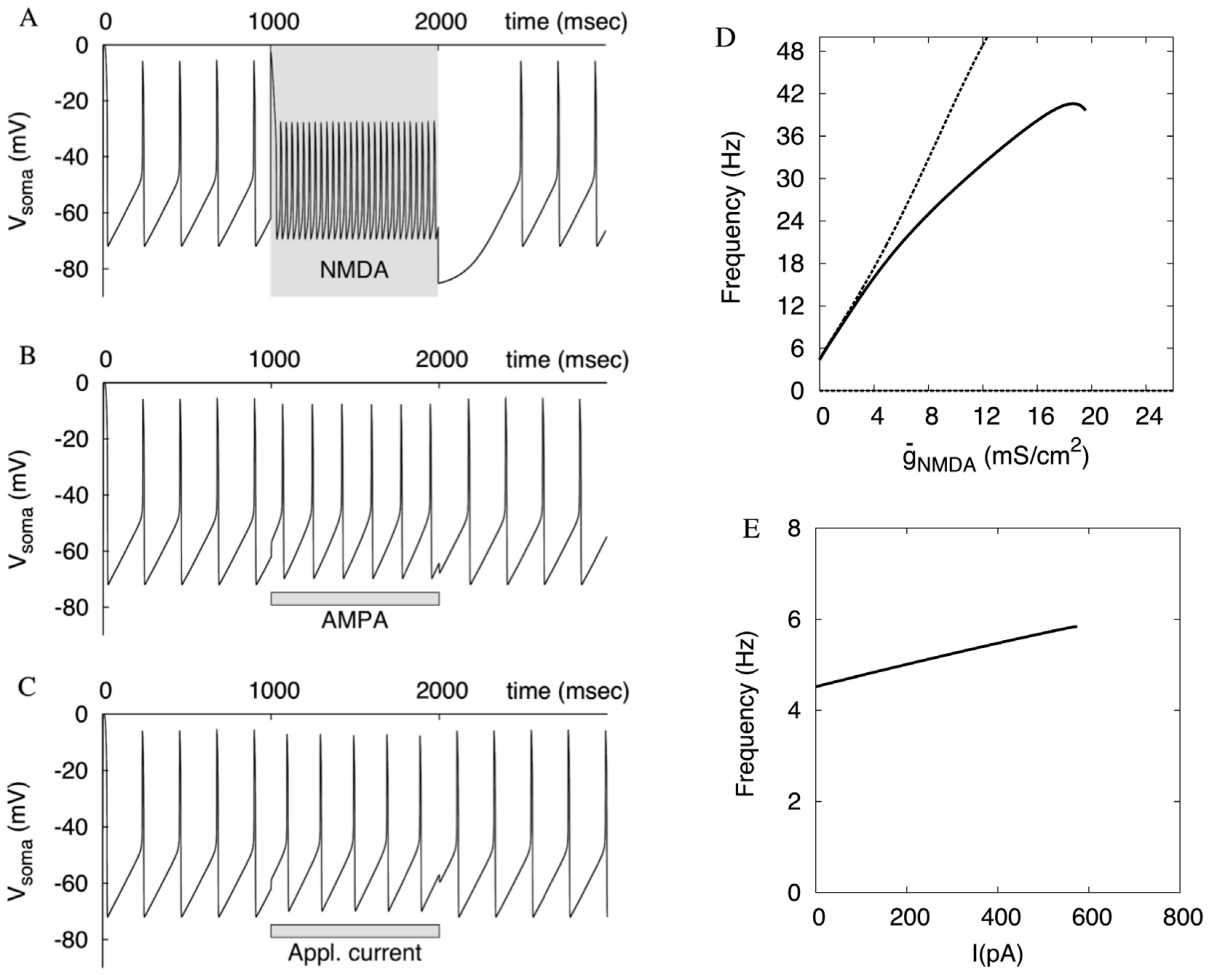
Typical responses of the one-compartmental model to NMDA, AMPA receptor stimulation and somatic depolarization are very close to those of the reconstructed morphology. As in Fig. 4, the values for the applied current and AMPAR stimulation are chosen slightly lower than those causing blockade of oscillations. (D) The frequency as a function of NMDAR conductance in the one-compartment model (compare with Fig. 2D). The solid curve is for spiking model. It is truncated at a sharp transition to subthreshold oscillations. The dashed curve is for the model with no spikes. The frequency continues to grow until oscillations are suppressed around 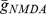 = 26 mS/cm^2^. (E) The frequency as a function of the applied current in the single-compartment nonspiking model. Oscillations are suppressed approximately at I = 580 pA.

**Figure 8:**
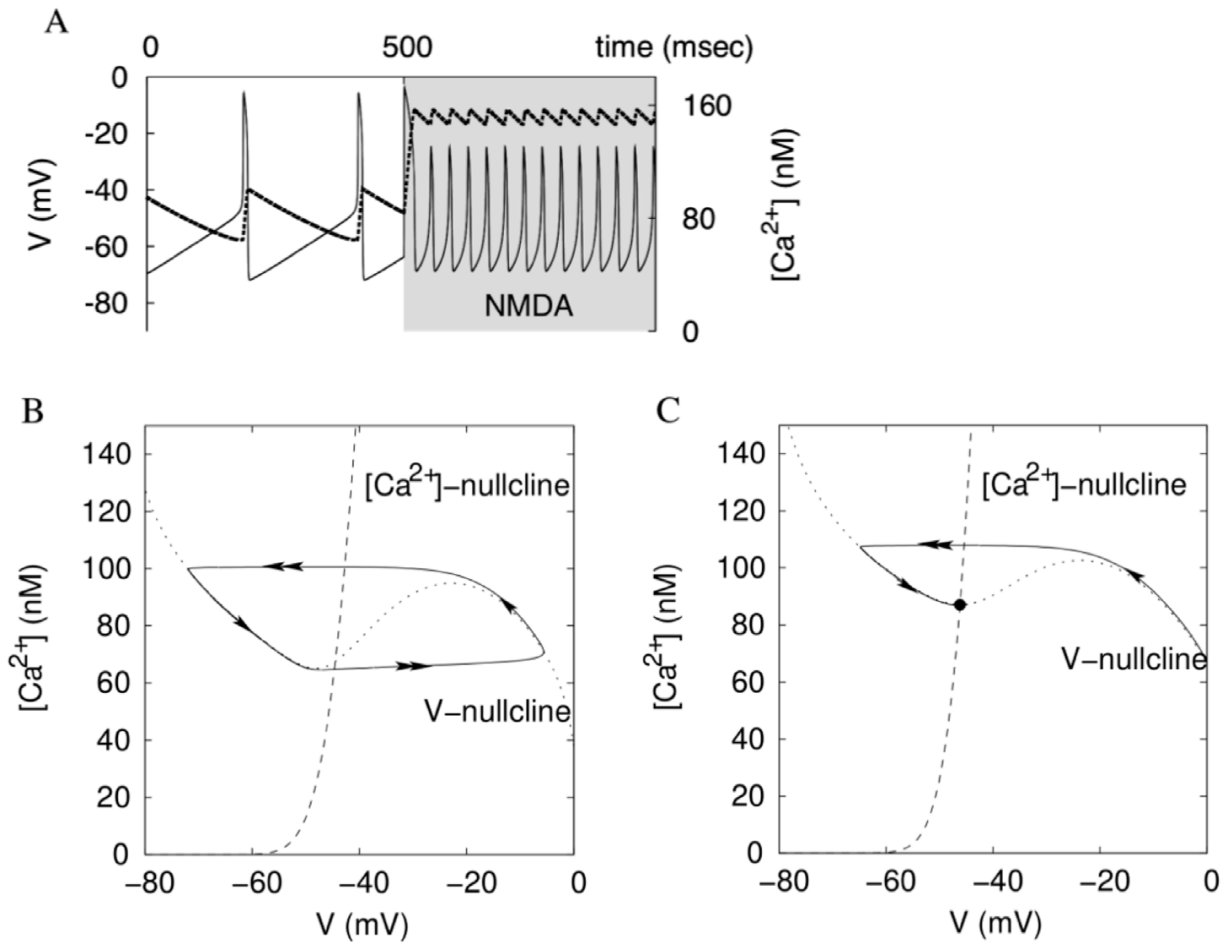
Oscillations of the voltage and Ca^2+^ concentration. (A) At the onset of high-frequency oscillations, the amplitude of Ca^2+^ concentration is dramatically reduced (dashed: Ca^2+^ concentration; solid: the voltage). (B) Oscillations are presented by a closed loop in the Ca^2+^-V plane. The oscillations circumscribe folding of the voltage nullcline (dotted). (C) The oscillations are blocked if the intersection of the voltage and Ca^2+^ nullclines interrupts the loop.

**Figure 9:**
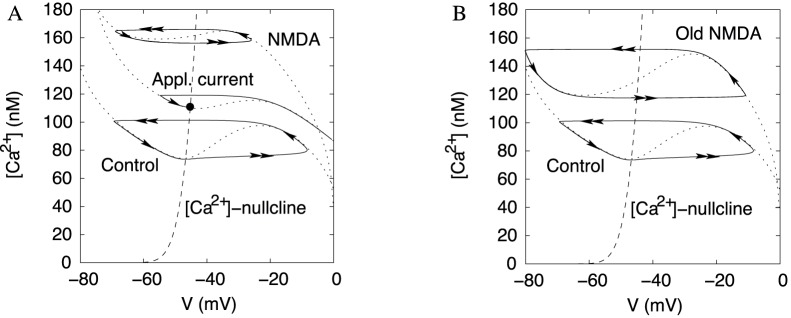
Changes in the voltage nullcline caused by the increasing applied current and maximal NMDAR conductance. (A) During applied depolarization, an equilibrium state interrupts the oscillatory cycle. During NMDAR activation, the voltage nullcline becomes more flat, and this causes a frequency increase. (B) If the NMDAR has a steeper voltage dependence, the voltage nullcline remains strongly folded, and the frequency remains low.

In the DA neuron, the oscillations persist in the subthreshold range after the blockade of the spike-producing currents (TTX application). Many publications document this for the low-frequency background activity (see e.g. [20]), and this was recently extended to the NMDA-evoked high-frequency activity [9]. Fig. 2D also shows the frequency responses during simulated blockade of the spike-producing fast sodium current (thin curves). The amplitude of oscillations becomes much smaller (see Figs. 4–9), whereas the frequency does not change significantly in the model. These matching frequencies in the model with and without the spiking currents mirror those in experimental data for TTX vs. control [9], [20]. The model does not allow for the comparison of the regularity of spiking and subthreshold oscillations [17] because no noise is included and the oscillations are perfectly regular. The match between spiking and subthreshold oscillations justifies our first reduction: in the rest of the manuscript, we show results for the model with no sodium spike-producing currents. The nonspiking model has the minimal number of biophysical components and the minimal number of variables (three: *v*, [Ca^2+^] and *n*) that are required for the described phenomena.

#### Tonic AMPAR stimulation and applied depolarization lead to early blockade of oscillations

By contrast to NMDA, applied depolarization or tonic AMPAR synaptic stimulation is unable to elevate the frequency in the DA neuron to the levels observed during bursting [9], [10], [11]. In our model, tonic applied depolarization elevates the frequency to 6 Hz only and then leads to blockade of oscillations (Fig. 4 A, C). Tonic activation of the AMPAR current also led to blockade of oscillations rather than a frequency increase (Fig. 4 B, D). The range of the AMPAR conductance where oscillations persist is strikingly smaller than that for the NMDAR conductance. Note that complex, mixed-mode-type oscillations are displayed during and after AMPAR stimulation. This is very similar to voltage traces obtained after the stimulation in experiments ([9], Fig. 4A) Therefore, the frequency remains limited below 10 Hz under both AMPAR activation and applied depolarization, whereas NMDAR activation breaks this frequency limitation.

#### A combination of calcium- and voltage-dependent currents repolarizes the cell

Two lines of evidence suggest that the fast currents hyperpolarizing after a spike (fast AHP complex), including the delayed rectifier potassium current, are weak in the DA neuron. First, our modeling shows that applied depolarization would evoke much higher frequencies if the delayed rectifier was stronger. This would erase the difference between the responses to AMPAR and NMDAR synaptic stimulations. Second, spiking becomes unreliable and the neuron becomes prone to the cessation of oscillations when the SK-type calcium-dependent potassium current is blocked pharmacologically. The blockade was used *in vitro* to elicit bursting [34], but within or without the bursts, spiking quickly collapsed to a plateau.

On the other hand, the neuron demonstrates calcium-independent firing, as we mentioned in the Introduction. This requires a calcium-independent repolarizing mechanism. We have introduced a voltage-dependent potassium current to play this role. The calibration of the current resulted in the half-activation around −50 mV, slow activation (∼62 ms) and even slower deactivation (∼362 ms). The slowness of the current, and especially that its deactivation is much slower than activation, suggests the ERG current as the best candidate [35]. The current sustains pacemaking in the absence of the SK current, but only in a very small interval of applied depolarization (Fig. 5 A&B). We found that this property is achieved by a close match between the half-activations of the Ca^2+^ current and our ERG current in the model. Our nullcline analysis below explains this connection. The slowness of the current determines that only moderate increase in the frequency of background firing is observed upon the blockade of the SK current (Fig. 5A), which matches experimental data [36], [37]. The difference between the activation and deactivation timescales allows the ERG current to maintain the voltage waveform with a narrow peak and slow release from the hyperpolarized state. Additionally, we slow down the activation of the current to make it negligible in the presence of the SK current (Fig. 6). This makes the SK current dominant in the oscillatory cycle. Upon the blockade of the SK current, the switch from it to the ERG current determines the narrowing range of applied depolarization where the neuron sustains oscillations.

Our simulations predict that the NMDA-evoked high-frequency oscillations are robust when the SK current is blocked. This is in contrast to the property that oscillations become even more fragile in response to applied depolarization (Fig. 5F). Oscillations persist in a wide range of NMDAR conductance density and display elevated frequencies (Fig. 5C,E). Moreover, moderate NMDAR stimulation allows oscillations to persist at much higher applied depolarization and rescue the neuron form the cessation of oscillations (Fig. 5D). This prediction extends the observation that NMDA-evoked high-frequency firing persists after blockade of the SK current by apamin [9]. In Supplement S1, we illustrate both low and high- frequency firing during the blockade of the SK current in the full model with spike-producing currents (Fig. S1B in Supplement S1). Thus the ERG current supports and amplifies the distinction between the responses to applied depolarization and NMDAR stimulation.

### One-compartmental Model

#### Minimal representation of the DA neuron

Next we show that a one-compartment representation is sufficient for reproducing distinct frequencies of subthreshold oscillations evoked in response to NMDA vs. AMPA receptor stimulation and applied depolarization. The above model implements minimal biophysical properties, but the number of compartments representing the reconstructed morphology makes it very complex. A reduction is essential for explaining our results. If all parts of the neuron are in synchrony, a one-compartment model will work. Fig. 7 shows typical responses of the one-compartment model to AMPAR and NMDAR activation and somatic depolarization. The results of stimulations closely match those for the reconstructed morphology and justify the assumption of synchrony (we compare the single compartment to the bath application of the agonists above). Now the radius of the compartment in Eq. (2) does not correspond to any particular part of the neuron any more. It represents an “average” compartment because all properties, including the frequency are effectively averaged by the coupling when the compartments are in synchrony. By choosing a value for the radius, we calibrated the model to reproduce maximal frequencies similar to those obtained during applied depolarization and NMDAR activation in experiments. Interestingly, the radius takes the value of 0.65 µm, which corresponds to fine distal dendrites. To understand this, let’s think of a reduced model with the same number of compartments, but where the radii of compartments are all the same and have the diameter of a distal dendrite. Thus, we replaced all proximal dendrites and the soma with distal dendrites. We know that such compartments synchronize as one and produce a mode similar to the reconstructed morphology. This suggests that the total contribution of the distal dendrites to the overall transmembrane current is very substantial, and distal dendrites control the frequency of the whole neuron. Only when there is a stimulus that splits the dendritic tree, the soma and proximal dendrites receive the opportunity to contribute more. The single-compartment model does not account for such cases, but it reproduces all other properties described above.

#### Mechanism for the high frequency: flattening the voltage nullcline

To explain the mechanism underlying the frequency responses, we simplify the model by blocking our ERG current. As a result, the model is reduced to a system of two variables: the voltage and calcium concentration (Eq. 1&2). Fig. 8A shows time series for these two variables at the onset of NMDA-evoked high-frequency oscillation. To show a good correspondence with the reconstructed morphology model that includes the spike-producing currents, we show the same transition in Fig. S1A of Supplement S1. In Fig. 8A, at the onset, the amplitude of Ca^2+^ oscillations is dramatically reduced. This underlies the frequency increase. A less steep voltage dependence of the NMDA current compared to the Ca^2+^ current determines the decrease in the amplitude of Ca^2+^ concentration. To explain this, we introduce a simple mathematical tool.

A basic mathematical formalism allows for geometric visualization of the changes introduced by NMDAR activation. Plotting the voltage and Ca^2+^ concentration against each other at every instance of time results in a cycle that represent oscillations (Fig. 8B). We investigate the dynamics by plotting *nullclines* of the system. These are two curves on which either Ca^2+^ or voltage equilibrates (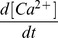 = 0 or 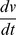 = 0, respectively). Oscillations circumscribe folding of the voltage nullcline as shown in Fig. 8B. The intersection of the two nullclines is an equilibrium state of the model. This state corresponds to the blockade of oscillations. It always exists, but may be unstable (repulsive) and, consequently, not displayed by the neuron. The oscillations are impossible if the equilibrium state interrupts the oscillatory cycle (Fig. 8C).

The voltage dependence of the NMDAR current is central to shaping the voltage nullcline. The changes to the nullclines under increasing maximal NMDAR current density and applied depolarization are shown in Fig. 9A. During NMDAR activation, the intersection of the nullclines remains between the extrema and remains an unstable equilibrium. During a current injection, the minimum of the voltage nullcline shifts across the calcium nullcline. As a result, the equilibrium state at their intersection interrupts the oscillatory cycle, and oscillations die out. The equilibrium becomes stable (attractive). This explains why, oscillations die out with a small elevation in the applied current, but not at the higher NMDAR current density.

Additionally, the NMDAR activation makes the voltage nullcline more flat. More precisely, the extrema become significantly closer to each other along the Ca^2+^ concentration axis, whereas they stay well separated along the voltage axis (Fig. 9A). Oscillations span the interval of the Ca^2+^ concentration between the extrema. The length of this interval is the primary determinant of the period of oscillations because Ca^2+^ concentration changes slowly. NMDAR shortens the interval of Ca^2+^ concentration between the extrema. As a result, NMDAR significantly reduces the period by reducing the amplitude of Ca^2+^ oscillations. Taken together, the high-frequency oscillations are achieved by flattening, but not unfolding the voltage nullcline.

**Figure 10:**
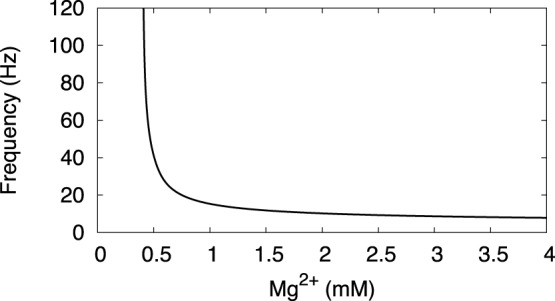
The dependence of the oscillation frequency on the magnesium concentration at a fixed NMDAR current density (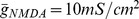).

Extracellular magnesium blocks the NMDA receptor at a low voltage. This defines the NMDAR current half-activation, and the higher the magnesium concentration, the higher the half activation. The dependence of the frequency on Mg^2+^ concentration was measured in dynamic clamp experiments [9]. Our model reproduces this dependence (Fig. 10). At a higher Mg^2+^ concentration, NMDAR current remains constantly blocked, and the frequency remains low. At a very low Mg^2+^ concentration, the NMDAR current is always open and looses its voltage dependence. It acts as the AMPA receptor and suppresses oscillations. In the middle range, the frequency increases with decreasing Mg^2+^ concentration and reaches above 100 Hz in its maximum. The properties closely match the experimentally obtained dependence and expand it to the higher Mg^2+^ concentration. Note that in [9] a different coefficient was used in the formula for the NMDAR current dependence, and our Mg^2+^ concentration must be divided approximately by the factor of 3 to obtain the same scale.

The other parameter of the dependence, 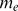 defines its slope. This slope determines how the voltage nullcline transforms as the NMDAR current is introduced. The slope of the voltage nullcline is directly related to the slope of the I–V curve for the neuron. In particular, a negative resistance caused by regenerative opening of depolarizing channels corresponds to the middle part of the voltage nullcline, where it has a positive slope. This part is steeper if the activation curves for the currents are steeper. Conversely, flattening of the voltage nullcline (Fig. 9A) reflects a gradual voltage dependence of the NMDAR current (Fig. 1B). The increase in the frequency corresponds to the gradual flattening of the voltage nullcline as the NMDAR contributes a greater portion of the depolarizing current. For comparison, Fig. 9B shows the nullclines for the NMDA current with a steeper voltage dependence (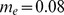). The vertical distance between the extrema of the voltage nullcline remains large. Accordingly, the frequency of oscillations remains low for any conductance of the NMDAR current. Therefore, the ability of the NMDAR current to evoke the high frequencies is linked to the moderate slope of its voltage dependence. This gives an easily testable prediction: a virtual NMDA current with a steeper voltage dependence will not elevate the frequency.

#### Dynamical consequences of combining the repolarizing currents

**Figure 11:**
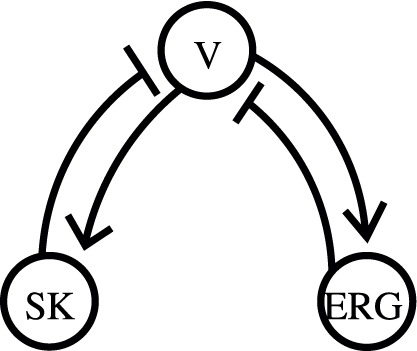
The structure of the model. Two negative feedback loops are interlocked by the voltage variable. Hammerheads show inhibition and arrows show activation.

The inclusion of the two repolarizing currents makes the model three-dimensional, where each of them is capable of sustaining oscillations. The currents form two negative feedback loops. They are shown in Fig. 11, where a hammerhead means inhibition and an arrow means activation. However, it’s impossible to simultaneously observe two different rhythms generated by the separate feedback loops. This is because the loops share a component, which is the voltage. Any manipulation that breaks a connection breaks at least one feedback loop. This distinguishes the structure of the model from coupled oscillators. Such structure is well known for the circadian clock and was called interlocked feedback loops.

**Figure 12:**
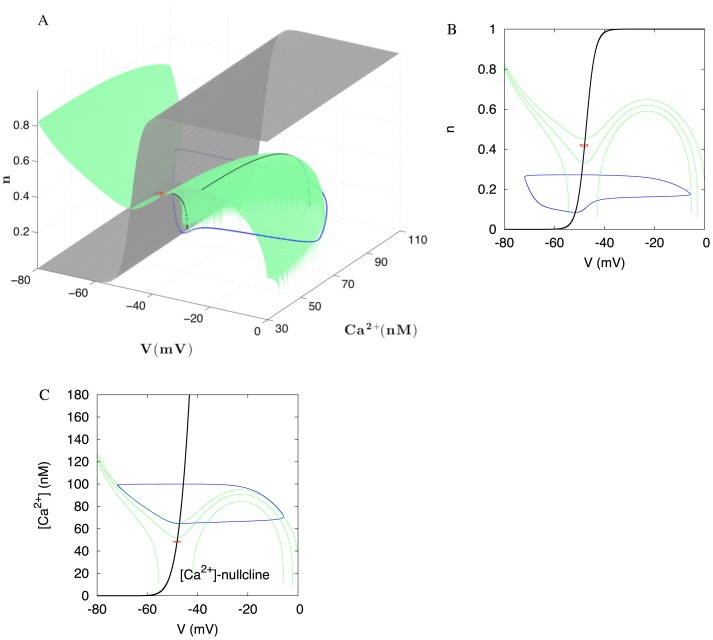
Three-dimensional structure of the model allows for complex modes and bistability. (A) Two simultaneously stable oscillatory solutions with very different amplitudes are shown in blue and red respectively. The voltage and n-nullclines extend onto null-surfaces. The Ca^2+^-nullcline is not shown for clarity. (B) and (C) Projections of the two solutions show their separation along Ca^2+^ concentration and the gating variable of the ERG current, n. Sections of the voltage null-surface are shown for a few values of the third variable: (B) [Ca^2+^]  = 30, 60, 70; (C) n = 0.1, 0.4, 0.5. The Ca^2+^ and n-nullclines (black) are the same for any value of the third variable.

Voltage oscillations of a complex form are often observed in experiments (see e.g. [9]), and our model suggests a way they can be generated. The one-compartment model displays bistability, in which stable oscillations coexist with a stable equilibrium state. In this case, the observation of oscillations or their blockade is dependent on the initial conditions (e.g. holding potential). Subtle differences in experiments may modulate initial conditions and lead to different coexisting modes. In Fig. 12A, two types of oscillations very different by their amplitude are observed in the model in the absence of stimulation. The high-amplitude oscillatory solution corresponds to the time series in Fig. 7 without stimulation (the first and the last second of each time-series). None of these stimulations switches the model into the low-amplitude mode shown in Fig. 12. Only after a stronger applied current or AMPAR stimulation that suppresses oscillations, the one-compartment model remains close to the equilibrium state and displays the low-amplitude oscillations. NMDAR stimulation also removes bistability, but switches the model into the high-amplitude mode. Interestingly, even after the suppression of oscillations achieved by excessive NMDAR stimulation, as soon as the stimulation ends, the model returns to the high-amplitude mode. The latter holds in the reconstructed morphology model (Fig. 2 C), but the model returns to the high-amplitude mode after suppression of oscillations by AMPAR stimulation or applied current, in contrast to the single compartment. Our simulations show different mixed modes and long complex transients after the stimulation (see Figs. 2 B&C, 4 A&B). We may view the mixed modes as switching between the high- and low-amplitude modes by interaction among compartments. Thus, we suggest that complex oscillatory modes in the reconstructed morphology are based on bistability in the minimal single-compartment model. Multiple mixed mode solutions coexist with each other. Thus, multystability and complex modes displayed in our model may explain apparently discrepant experimental results obtained for the DA neuron in different groups (e.g. [9], [11]).

## Discussion

### The Role of the DA Neuron Morphology

We have made two conclusions on the role of the DA neuron morphology: First, the morphology does not determine the capacity of the DA neuron to generate a high-frequency firing differentially in response to NMDAR, but not AMPAR stimulation or applied depolarization. Second, both the spread and the focus of NMDAR stimulation along dendrites affect the transition to the high-frequency firing. Both statements and a relation between them deserve attention.

Puzzling firing properties of the DA neuron trigger a search for very complex mechanisms, including its morphology and heterogeneity of membrane currents. In our previous study [12], the interaction of the dendrites and the soma determines the firing rate. However, this mechanism was disproved in experiments [9], [13]. The new property completely incompatible with the old mechanism was the ability of the focal somatic NMDA stimulation to elicit the high-frequency firing. This is the first property we have reproduced in our new model. The model is presented in two morphologies: (1) a reconstruction of a DA neuron, and (2) a single compartment that ignores the spatial structure of the neuron. A great correspondence between the two representations suggests that an unusual morphology of the DA neuron [38] is not necessary for the neuron to distinguish between NMDA and AMPA receptor stimulation. For this purpose, the whole dendritic tree may act as one compartment.

On the other hand, the dendritic morphology plays a role in synaptic integration of a heterogeneous stimulation. By the comparison of the two representations of our model, we made the conclusion that distal dendrites control the frequency of the neuron, but only when the dendrites are synchronized. A focal dendritic stimulation separates a part of the dendritic tree and impedes synchronization. In this case, the single compartment representation is not correct. The morphology determines the minimal portion of the dendritic tree that must receive NMDA stimulation to evoke high frequency firing. That was approximately three times greater area of distal dendrites compared to the soma in our model. By contrast, the somatic NMDAR stimulation spreads to all dendrites evenly and does not violate their synchronization. This leads to a surprising conclusion: the soma is at a much better location to manipulate the firing frequency, even though the frequency is controlled by distal dendrites.

### Multiple Pacemaking Mechanisms

A number of recent publications challenge the calcium-potassium pacemaking mechanism in the DA neuron and suggest Na^+^-based pacemaking instead [11], [16]–[18]. The Na^+^-based pacemaking requires a calcium-independent repolarizing current. Several fast voltage-gated hyperpolarizing currents have been identified in the neuron [39]. However, the fast AHP currents must be weak in he DA neuron because otherwise it would make the responses to AMPA and NMDA receptor activation indistinguishable and firing in the presence of apamin perfectly robust. Therefore, a slow voltage-sensitive hyperpolarizing current is necessary to sustain calcium-independent pacemaking, and no study suggests what current may play this role. We have introduced a simple voltage-dependent current and its calibration suggests the ERG current for this role. ERG1 proteins are expressed in the substantia nigra pars compacta neurons [23]. A slow AHP similar to that produced by the ERG current was measured in the DA neuron [24], although the current has not been fully characterized. Early studies of the ERG current showed its deactivation to be much slower than activation [35]. This is exactly what our calibration gives: the activation in the range of tens of msec. and deactivation in the range of hundreds of msec. Our half-activation of −47.4 mV closely match the experimental estimates. The slope of the activation function measured in Purkinje neurons is 5 mV, and we use 2 mV in our model. The difference may be explained by the variability of the current in different neuron types [35], [40]. The ERG current is suggested to be involved in termination of bursts [24], [41] and affect pacemaking in the DA neuron [25]. Our study attributes a new major role to this current for repolarizing in calcium-independent firing.

Oscillatory mechanisms in the DA cell have been intensely discussed. Originally, Ca^2+^ and Ca^2+^-dependent potassium currents were shown to sustain subthreshold oscillations as well as spiking in the DA neuron (see e.g. [19]–[21]). Then, a significant contribution of Na^+^ currents to pacemaking was shown [11], [16]–[18], [42], and the mechanism that generates spiking was proposed to be different from one generating the subthreshold oscillations [17]. Then the conclusion that the Ca^2+^ and Na^+^ currents contribute to the same oscillatory mechanism has been reached [22]. Thus, spike-producing currents alone are unable to sustain firing and require subthreshold drive, consistent with our conclusion above. However, this mechanism cannot sustain Ca^2+^-independent pacemaking found in the DA neuron [11], [16]–[18]. We propose the second subthreshold oscillatory mechanism that can play this role. The two mechanisms are defined by two negative feedback loops (Fig. 11). A Ca^2+^-dependent and a voltage-dependent repolarizing current constitute the loops, and each of the loops can sustain oscillations. The voltage-dependent loop represents the Ca^2+^-independent pacemaking. In our model, depolarization is still provided by the Ca^2+^ current, but once the SK current is blocked, the Ca^2+^ specificity of the current is irrelevant because Ca^2+^ concentration does not affect the voltage. In accordance with that, the voltage dependence, but not Ca^2+^ specificity of the Ca^2+^ current has been found critical for pacemaking [13]. Thus, our model supports the idea that there are multiple pacemaking mechanisms in the DA neuron. Our prediction is that the mechanisms must sustain subthreshold oscillations, which then drive spiking.

Another prediction can be tested in dynamic clamp experiments. Not only blockade of the ERG current, but also its enhancement does not significantly change the background firing in the presence of the SK current. When the SK current is blocked, our model predicts that a small enhancement of the ERG current blocks the oscillations. By contrast, a significant reduction of the ERG current (4.8 to 0.9 uS/cmˆ2) only changes the waveform: the voltage stays longer in the depolarized phase, which was observed in experiments and called plateau potentials [41]. Another predictions can be tested by a simple current clamp experiment when the ERG current is blocked and the SK current stays intact. Our simulations show that oscillations persist at a much higher applied depolarization (data not shown) in this case than when the ERG current is present. Our explanation is that, during applied depolarization, the ERG current develops a constant component that shunts the active currents. The mechanism is similar to shunting inhibition [43].

The structure of the model (Fig. 11) links firing patterns of the DA neuron with research in another branch of biology and may explain the role of this structure. Such structure defines oscillatory mechanisms that underlie the circadian rhythm [44]. This structure is called interlocked feedback loops. The loops are interlocked in the sense that it is impossible to separate them without losing the oscillations. This interlocked feedback loop structure has been suggested to improve robustness of the circadian rhythm [44]. Accordingly, blockade of the SK current in the DA neuron, which brakes one of the feedback loops, dramatically reduces robustness and regularity of firing [36], [37]. Interaction of multiple ion channels has been suggested to ensure robustness of pacemaking with respect to perturbations in individual components [17]. Robustness of oscillations can be studied using minimal models, in which biophysical details are omitted and only the structural properties are reflected. These models are the same for different applications, such as regulatory networks or electrical activity of a neuron. Therefore, the interlocked feedback loop representation provides an interdisciplinary framework for the analysis of robustness.

### The Role of NMDA in Eliciting Bursting

A number of studies associate bursting specifically with NMDA receptor activation both *in vivo* and *in vitro* [5], [7], [9], [45], [46]. The distinction between AMPA and NMDA receptor currents that allows the DA neuron to respond differentially is the NMDAR current voltage dependence [9], [13]. Our study supports the importance of the voltage dependence of the NMDAR current. We have shown that NMDAR and AMPAR activation elicit distinct responses because they interact differently with the mechanism for pacemaking in DA neurons. The NMDAR current expands the interval of negative resistance by its regenerative opening. As a result, NMDAR effectively counteracts decreasing amplitude of oscillations with respect to the voltage. Simultaneously, it significantly reduces the amplitude of the Ca^2+^ oscillations. This leads to a pronounced elevation of the frequency because Ca^2+^ concentration is a slow variable and its variations determine the period. This requires the slope of the voltage dependence of the NMDAR current to be moderate. We have shown that NDMAR current with a steeper voltage dependence never elevates the frequency. On the other hand, the NMDAR current with a gradual voltage dependence cannot restore regular pacing when the Ca^2+^ current is blocked in the model, which replicates experiments with nimodipine-silenced DA neurons [13]. The ability of the Ca^2+^ current, but not NMDA receptor current to sustain pacemaking was attributed to a difference in their half-activation. We stress that the slope of the voltage dependence plays even more important role: the frequency elevation would not be possible with a steeper dependence at any half-activation. Therefore, the model predicts that the ability of the NDMAR current to evoke the high frequencies is determined by the moderate slope of its voltage dependence.

Our results do not exclude the involvement of AMPA receptors with bursting in DA neurons. Previous experimental [11] and modeling [47] studies show that both AMPA and NMDA receptors contribute to burst firing. Here, the major distinction with our work is that the receptors were activated with high-frequency trains of electrical stimulation. Such stimulation evokes short powerful AMPA EPSPs by synchronous activation of the receptors. Our model is capable of following a pulsatile AMPA stimulation, but shows that stronger bursting results from an NMDA stimulation of the same intensity (Fig. S2 of Supplement S1). Similarly, pulses of applied depolarizing current at 25 Hz separated by weak hyperpolarizations evoke spiking at this frequency [9]. Our model replicates this experimental finding (data not shown). An asynchronous AMPA tone that sums into a plateau potential fails to elevate the frequency [9]. This suggests that in the *in viv*o conditions of the previously published studies [5]–[7] AMPA receptors activate asynchronously.

Our simulations show that NMADR stimulation dramatically increases the basal level of Ca^2+^ concentration (see Fig. 8A). This increase is due to the opening of the calcium current that follows depolarization. The flux of calcium via L-type Ca^2+^ channel is proposed to be responsible for the oxidative stress in the DA neuron and cause degeneration of the neurons leading to Parkinson disease [16]. Calcium influx through NMDA receptors will also contribute to the neurotoxicity [48]. Thus, our study suggests a pivotal role of NMDAR activation in degeneration of DA neurons.

### Connections to other Modeling Studies

The electrophysiology of the DA neuron has been modeled by several groups, and each model was focused on a particular property. The calcium-potassium mechanism for background firing was first modeled in a single compartment by [49], and then extended throughout the length of the dendrites by [20]. This gave rise to a coupled oscillator representation of the DA neuron. Here, we start with this representation and show that it can be reduced back to a single compartment.

Rhythmic bursting evoked by bath application of NMDA agonist in DA neurons was modeled in a number of studies [29], [50], [51]. These studies were focused on a specific feature for this type of bursting - the rhythmic plateau potentials that underlie bursts. As we emphasize above, the high-frequency firing cannot be a simple consequence of a plateau potential in DA neurons, and the present study offers a unique mechanism for the intraburst firing, which can be combined with plateau potential generation.

Bursting achieved by the blockade of the SK type Ca^2+^–dependent potassium current was modeled in a number of papers [22], [41], [47], [49], [52]. The ERG current was suggested to provide extraburst hyperpolarization in the absence of the SK current [25], [41]. We show that, surprisingly, such a slow current can sustain fast firing within the bursts. A stronger activation of this current can also end the burst and provide extraburst pauses, as shown earlier. The intraburst high-frequency firing was assumed to be a simple consequence of the plateau potentials in most of these papers, except for two. [52] show clustering spikes into high-frequency bursts only in the presence of an SK current blocker. NMDA stimulation alone could not evoke bursting in their model, and our model covers this gap. The authors of [22] mostly focus on the combination of Na^+^ and Ca^2+^ depolarizing currents and managed to reconcile apparently discrepant experimental results on their contribution (see e.g. [17]). However, their model continues firing in the absence of the SK current due to additional Ca^2+^-dependent repolarization by the Ca^2+^ pump, whereas our model offers a Ca^2+^–independent repolarizing current, which works for Na^+^–based pacemaling. Both cases are possible, and which repolarizing mechanism works when the SK current is blocked is a question for future experiments. While [22] suggests that the amplitude of calcium oscillations increases upon blockade of the SK current, our study predicts that the whole range of calcium concentrations shifts up more significantly. Thus, using this prediction, a future Ca^2+^–imaging experiment will determine the mechanism of repolarization.

Finally, the mechanism of high-frequency oscillations presented here is similar to one presented in our recent publication [53], [54]. Here and in these publications, flattening of the voltage nullcline determines the frequency elevation. The major difference is that here we link this flattening to the gradual dependence of the NMDA current. The alternative way presented before was based on the stretching of the voltage nullcline along Ca^2+^ concentration by the nonlinear dependence of the SK current. This stretched shape was initially formed so that a stimulation could shift the range of Ca^2+^ concentration to a linear part of the SK current dependence, and the nullcline became compressed. The elevation of frequency obtained this way was more steep compared to one obtained here (Fig. 2). Interestingly, the function of the NMDAR current voltage dependence used in dynamic clamp experiments had the reduced slope that we use in this work [9], [13]. This favors the mechanism presented here, but does not exclude the possibility that both mechanisms are combined in the neuron.

## Supporting Information

Supplement S1 (DOC)

Table S1 List of model parameters.
(DOC)

## References

[1] Carlson N (1999) Foundations of Physiological Psychology, 4th ed. Allyn and Bacon, MA.

[2] Wise R (2004) Dopamine, learning and motivation. Nature Rev Neuroscience 5: 483–494. 15152198 10.1038/nrn1406

[3] Strange P (2001) Antipsychotic drugs: importance of dopamine receptors for mechanisms of therapeutic actions and side effects. Pharmacol Rev 53: 119–133. 11171942

[4] Schultz W (2002) Getting formal with dopamine and reward. Neuron 36: 241–263. 12383780 10.1016/s0896-6273(02)00967-4

[5] Chergui K, Charlety PJ, Akaoka H, Saunier CF, Brunet JL, et al (1993) Tonic activation of NMDA receptors causes spontaneous burst discharge of rat midbrain dopamine neurons in vivo. Eur. J. Neurosci 5: 137–144. 10.1111/j.1460-9568.1993.tb00479.x 8261095

[6] Overton P, Clark D (1997) Burst firing in midbrain dopaminergic neurons. Brain Res Reviews 25: 312–334. 10.1016/s0165-0173(97)00039-8 9495561

[7] Tong ZY, Overton PG, Clark D (1996) Antagonism of NMDA receptors but not AMPA/kainate receptors blocks bursting in dopaminergic neurons induced by stimulation of the prefrontal cortex. J. Neural Transm. 103: 889–904. 10.1007/BF01291780 9013383

[8] Morikawa H, Khodakhah K, Williams J (2003) Two intracellular pathways medicate metabotropic glutamate receptor-induced ca2+ mobilization in dopamine neurons. J Neuroscience 23: 149–157. 10.1523/JNEUROSCI.23-01-00149.2003 PMC1408315 12514211

[9] Deister CA, Teagarden MA, Wilson CJ, Paladini CA (2009) An intrinsic neuronal oscillator underlies dopaminergic neuron bursting. J Neuroscience 50: 15888–97. 10.1523/JNEUROSCI.4053-09.2009 PMC2824818 20016105

[10] Richards C, Shiroyama T, Kitai S (1997) Electrophysiological and immunocytochemical characteristics of gaba and dopamine neurons in the substantia nigra of the rat. Neuroscience 80: 545–557. 9284356 10.1016/s0306-4522(97)00093-6

[11] Blythe SN, Wokosin D, Atherton JF, Bevan MD (2009) Cellular mechanisms underlying burst firing in substantia nigra dopamine neurons. J Neuroscience 49: 15531–15541. 10.1523/JNEUROSCI.2961-09.2009 PMC2834564 20007477

[12] Kuznetsov A, Kopell N, Wilson C (2006) Transient high-frequency firing in a coupled oscillator model of the mesencephalic dopaminergic neuron. J Neurophysiol 95: 932–937. 16207783 10.1152/jn.00691.2004

[13] Putzier I, Kullmann PH, Horn JP, Levitan ES (2009) Cav1.3 channel voltage dependence, not Ca^2+^ Selectivity, drives pacemaker activity and amplifies bursts in nigral dopamine neurons. J Neuroscience 49: 15414–15419. 10.1523/JNEUROSCI.4742-09.2009 PMC2796195 20007466

[14] Yung WH, Hausser MA, Jack JJ (1991) Electrophysiology of dopaminergic and non- dopaminergic neurones of the guinea-pig substantia nigra pars compacta in vitro. J. Physiol. (Lond.) 436: 643–667. 2061849 10.1113/jphysiol.1991.sp018571 PMC1181526

[15] Nedergaard S, Greenfield SA (1993) Sub-populations of pars compacta neurons in the substantia nigra: the significance of qualitatively and quantitatively distinct conductances. J. Neuroscience 48(2): 423–437. 10.1016/0306-4522(92)90502-s 1603327

[16] Chan CS, Guzman JN, Ilijic E, Mercer JN, Rick C, et al (2007) ‘Rejuvenation’ protects neurons in mouse models of Parkinson’s disease. Nature 447: 1081–1086. 17558391 10.1038/nature05865

[17] Guzman JN, Sánchez-Padilla J, Chan CS, Surmeier DJ (2009) Robust pacemaking in substantia nigra dopaminergic neurons. J Neuroscience 29: 11011–11019. 10.1523/JNEUROSCI.2519-09.2009 PMC2784968 19726659

[18] Khaliq ZM, Bean BP (2010) Pacemaking in dopaminergic ventral tegmental area neurons: depolarizing drive from background and voltage-dependent sodium conductances, Journal of Neuroscience 30(21), 7401–13. 10.1523/JNEUROSCI.0143-10.2010 PMC2892804 20505107

[19] Grace AA, Onn S-P (1989) Morphology and electrophysiological properties of immunocytochemically identified rat dopamine neurons recorded in vitro. J. Neurosci. 9: 3463–3481. 10.1523/JNEUROSCI.09-10-03463.1989 PMC6569889 2795134

[20] Wilson CJ, Callaway JC (2000) A coupled oscillator model of the dopaminergic neuron of the substantia nigra. J Neurophysiology 83: 3084–3100. 10.1152/jn.2000.83.5.3084 10805703

[21] Wolfart J, Roeper J (2002) Selective coupling of T-type calcium channels to SK potassium channels prevents intrinsic bursting in dopaminergic midbrain neurons. J. Neurosci. 223404–3413. 10.1523/JNEUROSCI.22-09-03404.2002 PMC6758365 11978817

[22] Drion G, Massotte L, Sepulchre R, Seutin V (2011) How Modeling Can Reconcile Apparently Discrepant Experimental Results: the Case of Pacemaking in Dopaminergic Neurons. PLoS Computational Biology 7(5): e1002050. 21637742 10.1371/journal.pcbi.1002050 PMC3102759

[23] Papa M, Boscia F, Canitano A, Castaldo P, Sellitti S, et al (2003) Expression pattern of the ether-a-go-go-related (ERG) K^+^ channel-encoding genes ERG1, ERG2, and ERG3 in the adult rat central nervous system. J Comp Neurol 466: 119–135. 14515244 10.1002/cne.10886

[24] Nedergaard S (2004) A Ca^2+^??-independent slow afterhyperpolarization in substantia nigra compacta neurons. Neuroscience 125: 841–852. 15120845 10.1016/j.neuroscience.2004.02.030

[25] Ji H, Tucker KR, Putzier I, Huertas MA, Horn JP, et al (2012) Functional characterization of ether-à-go-go-related gene potassium channels in midbrain dopamine neurons - implications for a role in depolarization block. Eur J Neurosci. 36(7): 2906–16. 10.1111/j.1460-9568.2012.08190.x PMC4042402 22780096

[26] Durante P, Cardenas CG, Whittaker JA, Kitai ST, Scroggs RS (2004) Low-threshold L-type calcium channels in rat dopamine neurons. J Neurophysiol 91: 1450–1454. 14645383 10.1152/jn.01015.2003

[27] Helton TD, Xu W, Lipscombe D (2005) NeuronalL-typecalciumchannels open quickly and are inhibited slowly. J Neuroscience 25: 10247–10251. 10.1523/JNEUROSCI.1089-05.2005 PMC6725800 16267232

[28] Kohler M, Hirschberg B, Bond CT, Kinzie JM, Marrion NV, et al (1996) Small-conductance, calcium-activated potassium channels from mammalian brain. Science 273: 1709–1714. 8781233 10.1126/science.273.5282.1709

[29] Li YX, Bertram R, Rinzel J (1996) Modeling N-methyl-D-aspartate-induced bursting in dopamine neurons. Neuroscience 71: 397–410. 9053795 10.1016/0306-4522(95)00483-1

[30] Jahr CE, Stevens CF (1990) Voltage Dependence of NMDA-Activated Macroscopic Conductances Predicted by Single-Channel Kinetics. J. Neurosci. 10(9): 3178–3182. 10.1523/JNEUROSCI.10-09-03178.1990 PMC6570236 1697902

[31] Holmes WR, Levy WB (1990) Insights into associative long-term poten- tiation from computational models of NMDA receptor-mediated calcium influx and intracellular calcium concentration changes. *J. Neurophysiol.* . 63: 1148–1168. 10.1152/jn.1990.63.5.1148 2162921

[32] Ermentrout B (2002) Simulating, analyzing, and animating dynamical systems, SIAM, PA.

[33] Kuznetsova AY, Huertas M, Kuznetsov AS, Papadini C, Canavier C (2010) Regulation of firing frequency in a computational model of a midbrain dopaminergic neuron. J. Comp. Neuroscience 28(3): 389–403. 10.1007/s10827-010-0222-y PMC2929809 20217204

[34] Shepard P, Bunney B (1991) Repetitive firing properties of putative dopamine-containing neurons in vitro: regulation by an apamin-sensitive Ca^2+^-activated K^+^ conductance. Exp Brain Res 86: 141–150. 1756785 10.1007/BF00231048

[35] Sanguinetti M, Jurkiewicz N (1990) Two components of cardial delayed rectifier K^+^ current. J. Gen. Physiol. 96: 196–215. 10.1085/jgp.96.1.195 PMC2228985 2170562

[36] Nedergaard S, Flatman JA, Engberg I (1993) Nifedipine- and omega-conotoxin-sensitive Ca^2+^ conductances in guinea-pig substantia nigra pars compacta neurones. J. Physiol. (Lond.) 466: 727–747. 8410714 PMC1175500

[37] Ji H, Shepard PD (2006) SK Ca^2+^ -activated K^+^ channel ligands alter the firing pattern of dopamine-containing neurons in vivo. J. Neuroscience 140: 623–633. 10.1016/j.neuroscience.2006.02.020 16564639

[38] Juraska JM, Wilson CJ, Groves PM (1977) The substantia nigra of the rat: a Golgi study. J Comp Neurol 172: 585–600. 65369 10.1002/cne.901720403

[39] Silva NL, Pechura CM, Barker JL (1990) Postnatal rat nigrostriatal dopaminergic neurons exhibit five types of potassium conductances. J. Neurophysiol. 64(1): 262–272. 10.1152/jn.1990.64.1.262 2388070

[40] Sah P, McLachlan EM (1992) A slow voltage-activated potassium current in rat vagal neurons. Proc. Biol. Sci. 249(1324): 71–76. 10.1098/rspb.1992.0085 1359551

[41] Canavier CC, Oprisan SA, Callaway JC, Ji H, Shepard PD (2007) Computational Model Predicts a Role for ERG Current in Repolarizing Plateau Potentials in Dopamine Neurons: Implications for Modulation of Neuronal Activity J Neurophysiol. 98: 3006–3022. 10.1152/jn.00422.2007 17699694

[42] Puopolo M, Raviola E, Bean BP (2007) Roles of subthreshold calcium current and sodium current in spontaneous firing of mouse midbrain dopamine neuron. J. Neuroscience 27: 645–656. 10.1523/JNEUROSCI.4341-06.2007 PMC6672803 17234596

[43] Alger BE, Nicoll RA (1979) GABA-mediated biphasic inhibitory responses in hippocampus. Nature 281: 315–317. 551280 10.1038/281315a0

[44] Glossop NR, Lyons LC, Hardin PE (1999) Interlocked feedback loops within the Drosophila circadian oscillator. Science 286 (5440): 766–8. 10.1126/science.286.5440.766 10531060

[45] Johnson SW, Seutin V, North RA (1992) Burst firing in dopamine neurons induced by N-methyl-D-aspartate: role of electrogenic sodium pump. Science 258: 655–657. 10.1126/science.1329209 1329209

[46] Johnson S, Wu Y (2004) Multiple mechanisms underlie burst firing in rat midbrain dopamine neurons in vitro. Brain Res 1019: 293–296. 15306267 10.1016/j.brainres.2004.06.022

[47] Canavier CC, Landry RS (2006) An increase in AMPA and a decrease in SK conductance increase burst firing by different mechanisms in a model of a dopamine neuron in vivo. J Neurophysiol. 96(5): 2549–63. 10.1152/jn.00704.2006 PMC2531289 16885519

[48] Tymianski M, Charlton MP, Carlen PL, Tator CH (1993) Source specificity of early calcium neurotoxicity in cultured embryonic spinal neurons. J Neurosci. 13(5): 2085–104. 10.1523/JNEUROSCI.13-05-02085.1993 PMC6576557 8097530

[49] Amini B, Clark JW, Canavier CC (1999) Calcium dynamics underlying pacemaker-like burst firing oscillations in midbrain dopaminergic neurons: A computational study. J. Neurophysiol. 82: 2249–2261. 10.1152/jn.1999.82.5.2249 10561403

[50] Canavier CC (1999) Sodium dynamics underlying burst firing and putative mechanisms for the regulation of the firing pattern in midbrain dopamine neurons: a computational approach. J. Comput. Neurosci. 6: 49–69. 10.1023/a:1008809000182 10193646

[51] Komendantov AO, Komendantova OG, Johnson SW, Canavier CC (2004) A modeling study suggests complementary roles for GABAA and NMDA receptors and the SK channel in regulating the firing pattern in midbrain dopamine neurons. J. Neurophysiol. 91: 346–357. 10.1152/jn.00062.2003 13679411

[52] Oster A, Gutkin BS (2011) A reduced model of DA neuronal dynamics that displays quiescence, tonic firing and bursting. J Physiol Paris. 105(1–3): 53–8. 10.1016/j.jphysparis.2011.07.012 21939761

[53] Zakharov DG, Kuznetsov AS (2012) A minimal model for a slow pacemaking neuron, Chaos, Solitons & Fractals, 45, 640–644.

[54] Zakharov DG, Kuznetsov AS (2012) Mechanism of differentiation of neural responses to excitatory input signals, JEPT Letters, 95(11), 676–680.

