# Supplementary material for: Interaction of NMDA Receptor and Pacemaking Mechanisms in the Midbrain Dopaminergic Neuron
Source: PLoS One. 2013 Jul 19;8(7):e69984. doi: 10.1371/journal.pone.0069984 (PMC3716766; doi:10.1371/journal.pone.0069984)
Supplement: Supplement S1 — (DOC) [file pone.0069984.s001.doc]

Supplement

Because several critical properties of the model were illustrated only in the reduced model, we now show the same properties in the original model. The model includes the spike-producing sodium current and runs in the reconstructed morphology (Fig. 3A). Fig. S1 illustrates both the low frequency background firing and high-frequency firing evoked by NMDAR stimulation during the blockade of ether ERG or SK currents.


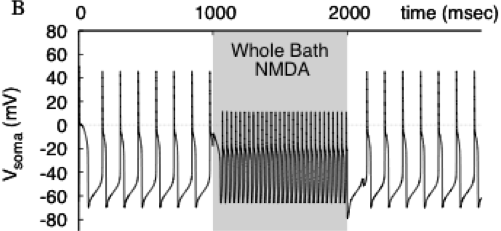

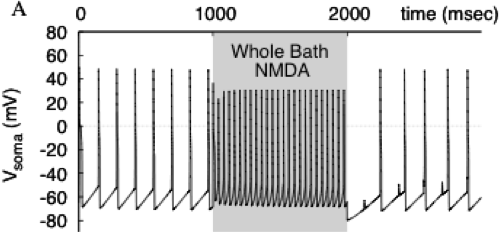


Fig. S1: Reconstructed morphology model with spikes retains the properties of the reduced interlocked feedback loop model. The low and high-frequency oscillations persist after (A) blockade of the ERG current; (B) blockade of the SK current.

All of the above simulations best reproduce the in vitro application of NMDAR or AMPAR agonists. In vivo, or for phasic high-frequency stimulation in slices, the pattern of stimulation may be very different: depending on how synchronous the inputs are, they can evoke pulses of receptor activation instead of summing up into a tonic activation level. Moreover, NMDAR current lasts much longer than AMPAR, which creates an additional difference with AMPAR current. We modeled the gating variables by a simple first order equation

where the subscript *i* stands for AMPA and NMDA respectively. We have chosen the opening and closing time-constants to be 1 and 5 ms for AMPAR and 3.3 and 200 ms for NMDAR. The parameters are within the range determined in experiments for the DA neuron (Gotz et al. 1997). The input *z* is a spike train with Poisson distribution and the mean frequency of 23 Hz. Figure S2 shows the time series for the synaptic variables and the voltage responses in three cases: activation of both AMPA and NMDA receptors, and each of them separately. The same seed for the stochastic variable was used so that the stimulation is exactly the same in all three cases. To mimic synaptic adaptation and other processes that make the synapse nonlinear, we used a sigmoidal function of the synaptic variable in the currents

with the same parameters for AMPA and NMDA receptors *s_th_*=0.06. *s_sl_*=0.01. Next, we estimate the burstiness of the time series (grouping of spikes in bursts). Our calculations show that the burst measure based on the classical burst definition by Grace and Bunney (1984) breaks down. This has been already observed (Oster and Gutkin 2011) and occurs because at higher average frequencies (11 to 16 Hz in our simulations) the definition registers almost all spikes being in bursts. An alternative measure of burstiness by van Elburg and van Ooyen (2004) has been used for the DA neuron model (Oster and Gutkin 2011). In our simulations, the measure has the lowest value for AMPAR stimulation alone (0.02), and the highest for the NMDAR stimulation alone (0.06), whereas the simultaneous stimulation of both receptors resulted in an intermediate burstiness (0.026). The values are low for all three cases because the bursts interrupt a lower frequency activity, and the majority of spikes are never grouped in bursts either in our simulations or in experiments (Grace and Bunney 1984). Thus, the simulations confirm the fact that the burstiness mostly depends on the NMDAR activation observed in experiments (Chergui et al., 1993; Overton and Clark, 1997; Tong et al., 1996).


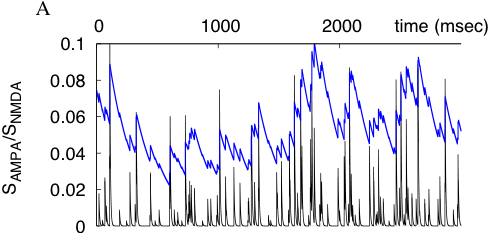

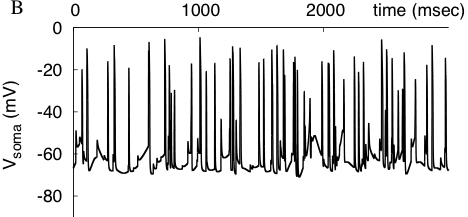

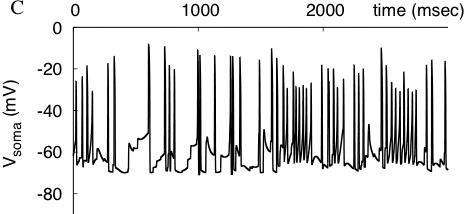

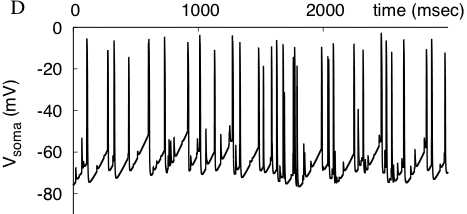


Fig. S2: Responses of the reduced model to realistic synaptic inputs. (A) Typical time series of the NMDAR (blue) and AMPAR (black) synaptic variables in response to a common glutamatergic input. (B) The voltage time series in response to both AMPAR and NMDAR stimulation. (C) Somewhat higher frequencies and apparent burstiness of the voltage oscillations is achieved when AMPAR is blocked. (D) By contrast, the frequency and burstiness are reduced if the NDMAR is blocked.

Supplement References

Gotz T, Kraushaar U, Geiger J, Lubke J, Berger T, Jonas P (1997) Functional properties of AMPA and NMDA receptors expressed in identified types of Basal Ganglia Neurons. J. Neuroscience, 17(1):204-215.

Grace, A.A., Bunney, B.S., 1984. The control of firing pattern in nigral dopamine 513 neurons: burst firing. J. Neurosci. 4, 2877–2890.

van Elburg, R.A.J., van Ooyen, A., 2004. A new measure for bursting. 559 Neurocomputing 58–60, 497–502.
