# Supplementary material for: Interaction of NMDA Receptor and Pacemaking Mechanisms in the Midbrain Dopaminergic Neuron
Source: PLoS One. 2013 Jul 19;8(7):e69984. doi: 10.1371/journal.pone.0069984 (PMC3716766; doi:10.1371/journal.pone.0069984)
Supplement: Table S1 — List of model parameters. (DOC) [file pone.0069984.s002.doc]

| Parameter | Meaning | Value |
| --- | --- | --- |
| $\bar{g}_{\text{AMPA}}$ | Maximal conductance of the AMPAR current | varies mS/cm^2^ |
| $\bar{g}_{\text{Ca}}$ | Maximal conductance of the calcium current | 2.5 mS/cm^2^ |
| $g_{L}$ | Maximal conductance of the leak current | 0.5 mS/cm^2^ |
| $\bar{g}_{K}$ | Maximal conductance of the potassium current | 1 mS/cm^2^ |
| $\bar{g}_{\text{ERG}}$ | Maximal conductance of the ERG current | 4.8 mS/cm^2^ |
| $\bar{g}_{\text{KCa}}$ | Maximal conductance of the SK current | 7.8 mS/cm^2^ |
| $\bar{g}_{\text{Na}}$ | Maximal conductance of the fast sodium current | 200 mS/cm^2^ |
| $\bar{g}_{\text{NMDA}}$ | Maximal conductance of the NMDAR current | varies mS/cm^2^ |
| k | Half-activation of the SK current | 160 nM |
| β | Buffering coefficient (ratio of free to total calcium) | 0.0003 |
| I | Applied current | varies pA |
| [Mg^2+^] | Magnesium concentration | 1.4 mM |
| P_Ca_ | Maximum calcium pump rate | 2500 μM/s |
| E_AMPA_ | Resting potential of the AMPAR current | 0 mV |
| E_Ca_ | Resting potential of the calcium current | 50 mV |
| E_L_ | Resting potential of the leak current | -35 mV |
| E_K_ | Resting potential of the potassium current | -90 mV |
| E_NMDA_ | Resting potential of the NMDAR current | 0 mV |
| c_m_ | Membrane capacitance | 1 μF/cm^2^ |
| A | Area of the neuron (for the single compartment) | 10^-4^ cm^2^ |
